# Spontaneous Bacterial Pericarditis and Coronary Sinus Endocarditis Caused by Oxacillin-Susceptible *Staphylococcus aureus*

**DOI:** 10.1155/2010/984562

**Published:** 2010-05-30

**Authors:** Maurício N. Machado, Marcelo A. Nakazone, Isabela T. Takakura, Carolina M. P. D. C. Silva, Lilia N. Maia

**Affiliations:** ^1^Department of Cardiology and Cardiovascular Surgery, São José do Rio Preto Medical School, CEP 15090.000, São José do Rio Preto, SP, Brazil; ^2^Department of Molecular Biology, São José do Rio Preto Medical School, CEP 15090.000, São José do Rio Preto, SP, Brazil; ^3^Intensive Care Unit, Heart Institute, School of Medicine, University of São Paulo, CEP 05403.900, São Paulo, SP, Brazil

## Abstract

This paper describes a case of a 44-year-old male patient previously healthy admitted with an unusual spontaneous acute bacterial pericarditis associated with coronary sinus mass. Two-dimensional echocardiography showed large loculated pericardial effusion with signs of diastolic restriction and an image suggesting vegetation in topography of the right atrium coronary sinus. Pericardial drainage, coronary sinus vegetation resection, and antibiotic therapy with Oxacillin were performed due to Oxacillin-susceptible *Staphylococcus aureus* identified on the pericardial effusion and blood culture. This is a rare condition and a unique combination of a spontaneous acute bacterial pericarditis with coronary sinus endocarditis without cardiac valve compromise.

## 1. Introduction

Thrombosis of the coronary sinus has rarely been described in the medical literature and may be associated with complications related to right heart catheterization, central venous access, and heart surgery [1–3]. Thrombophlebitis of the coronary sinus and spontaneous bacterial pericarditis are even rarer though most commonly found in severely ill and immunosuppressed patients with poor clinical evolution [4, 5].

The present paper describes the case of a previously healthy male patient suffering from spontaneous acute bacterial pericarditis caused by Oxacillin-sensitive *Staphylococcus aureus* and with a coronary sinus vegetative growth which was successfully treated by surgery and antibiotic therapy.

## 2. Case Report

A 44-year-old previously healthy male patient has presented with fever, dry cough, and dyspnea for 20 days. He was examined by a pulmonologist in the outpatients' clinic who requested a chest radiography which was unremarkable. The patient's symptoms were treated. Five days later, dyspnea and thoracic pain were worse and the patient returned to the pulmonologist who auscultated pericardial friction rub. The patient was refereed to hospital with the initial blood tests showing leukocytosis (19.4 × 10^9^/L) and an increase in the C-reactive protein 117.0 mg/L (reference value <10.0 mg/L). Two blood cultures identified Oxacillin-sensitive *Staphylococcus aureus*. Two-dimensional echocardiography showed moderate pericardial effusion with mobile mass adhered on the right atrium. Treatment with Oxacillin was started improving fever and white blood cell count.

The thyroid-stimulating hormone and free-thyroxine levels were normal. Tests for hepatitis B and C and HIV were negative. The screening for rheumatoid factor, antinuclear factor, and anti-DNA was nonreactant. The Mantoux test was negative. Thoracic computed tomography did not show intrathoracic masses or thoracic or hilar adenomegaly.

**Figure 1:**
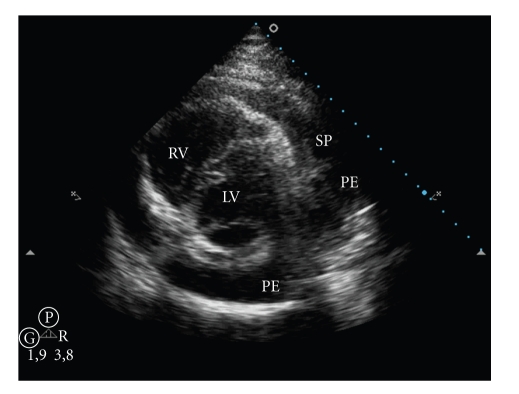
Transthoracic echocardiography: short axis view showing large pericardial effusion with septations (PE: Pericardial effusion; SP: septations; LV: left ventricle; RV: right ventricle).

**Figure 2:**
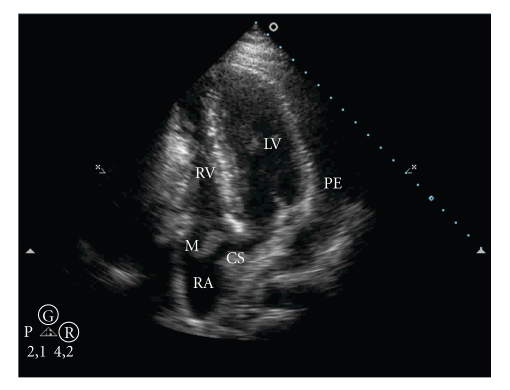
Transthoracic echocardiography: apical four-chamber view showing large pericardial effusion and mass in the right atrium at the coronary sinus (RA: right atrium; LV: left ventricle; RV: right ventricle; CS: coronary sinus; PE: Pericardial effusion; M: mass).

After 10 days of antibiotic therapy, new echocardiography showed large loculated pericardial effusion with signs of diastolic restriction (Figure 1) and a 4.0 × 0.5 cm echogenic coronary sinus mass on the right atrium (Figure 2). There were no alterations of cardiac valves, and left ventricular ejection fraction was normal (LVEF 73%).

**Figure 3:**
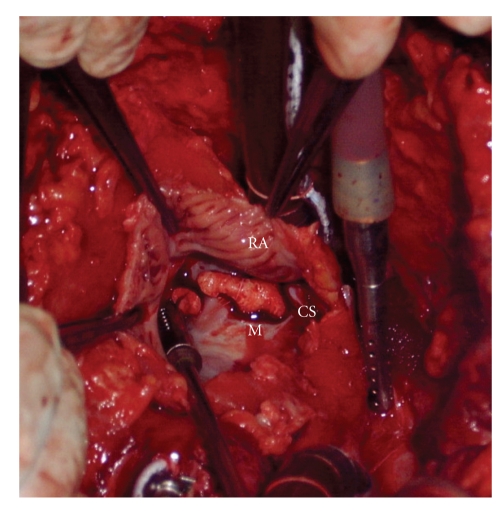
Right atriotomy showing the coronary sinus vegetation (RA: right atrium; CS: coronary sinus; M: mass).

Due to persistent dyspnea and lack of echocardiographic improvement the patient underwent cardiac surgery by median sternotomy draining a large quantity of purulent pericardial effusion. The vegetative growth adhered to the coronary sinus was excisioned (Figure 3). Bacterioscopic examination of the pericardial fluid showed gram-positive cocci, and Oxacillin-sensitive *Staphylococcus aureus* was isolated from a culture of the fluid. Due to failure in the material preparation, it was not possible to assess the gram or the appearance of the anatomo pathological sample. There were no signs of bacteria in the vegetation culture.

The postoperative period was event-free. A postoperative control echocardiography showed slight pericardial effusion with the coronary sinus patent and without apparent injury.

## 3. Discussion

Coronary sinus thrombosis is rare. Lesions associated with coronary sinus catheterization for retrograde cardioplegia during heart surgery with a cardiopulmonary bypass have been described in 0.095% of cases and are normally related to endothelial injury [2]. In procedures with access to the right atrium (including hemodynamic studies of the right heart chambers, central venous access, pacemakers, and cardiac resynchronization) which is necessary, the coronary sinus is more susceptible to accidental injury and subsequent thrombosis [1, 3, 6–8].

Infectious processes of the coronary sinus are even rarer. In 1976, Dryer et al. [4] described the case of a young patient with a history of drug abuse who went to hospital with septic shock caused by mitral valve bacterial endocarditis caused by *Staphylococcus aureus.* The necroscopic exam showed thrombophlebitis of the coronary sinus. Ross et al. [5] described the case of a patient with occlusion of the coronary ostia of the four large epicardial vessels and of the coronary sinus caused by *Aspergillus fumigatus* and also found *postmortem*. Another case describes diagnosis and treatment of septic thrombophlebitis in a patient who had previously underwent heart surgery (which may explain an injury in the coronary sinus because of a central venous access orundergoingcatheterization of the pulmonary artery).Clinically, the patient had repeated furuncles, probably because of bacteremia caused by *Staphylococcus aureus *[9].

Pericardium spontaneous bacterial infection is also uncommon due to its association with endocarditis because there is no relationship between pericardium venous drainage and the coronary system. Bacterial pericarditis normally occurs due to trauma, including thoracic surgery, pericardial, drainage, and propagation of an intrathoracic, myocardial, or subdiaphragmatic focus or hematogenic dissemination [10]. The mortality rate of patients with bacterial pericarditis is high. It may be as high as 40% of patients diagnosed and treated, and it may be up to 85% of nontreated patients [10].

What makes our case unusual is the fact that the patient, who had been healthy until that time, was under a clinical condition of acute pericarditis, severe pericardial effusion, and coronary sinus mass with intraoperative macroscopic vegetative aspect on the right atrium. However, the patient did not have cardiac valve compromise suggesting endocarditis. The patient had not been submitted to any previous procedures that could have led to trauma of the coronary sinus, predisposition to pericarditis or bacterial endocarditis. There were no signs of immunosuppression similar to those found in patients with neoplasias, autoimmune diseases, diseases of the conjunctive tissue, and infectious diseases like HIV.

Nevertheless, it is not possible to determine if the endocarditis of the coronary sinus allows hematogenic dissemination to the pericardium or vice versa.

This case represents the only description of a vegetative growth in the coronary sinus associated with spontaneous bacterial pericarditis caused by *Staphylococcus aureus* without cardiac valve infection, underwent pericardial drainage, removal of the vegetation from the coronary sinus, and antibiotic treatment.
